# The Rural Family Medicine Café Project: A Social Media Strategy to Reduce Occupational Isolation and Improve Support for Rural Healthcare Professionals

**DOI:** 10.3389/fpubh.2020.595255

**Published:** 2020-11-19

**Authors:** Amber Wheatley, Mayara Floss, Maria Bakola, Maria Kampouraki, Bianca Silveira, Jo Scott-Jones

**Affiliations:** ^1^Ysbyty Glan Clwyd, Betsi Cadwaladr University Health Board, Bodelwyddan, United Kingdom; ^2^Grupo Hospitalar Conceição, Porto Alegre, Brazil; ^3^Postgraduate Program of Public Health, University of Patras, Patras, Greece; ^4^16th Local Unit of Health, Kordelio-Evosmos, Thessaloniki, Greece; ^5^Escola de Saude Publica, Florianopolis, Brazil; ^6^Pinnacle Midlands Health Network, Hamilton, New Zealand

**Keywords:** occupational isolation, rural, social media, family medicine, rural training, rural education

## Abstract

**Background:** Globally rural medicine is currently suffering from staff shortages. Social and professional isolation are identified as significant pressures on health professionals working in rural areas. Social media (SOME) has created new methods of social engagement where conventional forms have failed. The Rural Family Medicine Café (RFMC) is a SOME project created to engage and support those interested in rural family medicine thus decreasing occupational isolation.

**Methods:** A quantitative analysis of SOME activity associated with the RFMC was done by measuring the frequency of #ruralcafe, #ruralwomenGP, #ruralGP, #ruralstories, and #ruralmedicine from October 2015 to October 2016 along with the number of Facebook page likes and YouTube views. A time series and regression analysis were done to assess the correlation between the frequencies of hashtag use and the number of new likes or views. A qualitative analysis of the content of tweets using the associated hashtags and comments on the RFMC YouTube videos was then done to assess participants' response to the RFMC. To add context to the data collected, regularly attending participants were invited for a semi-structured interview.

**Results:** There was a positive trend in the number of Facebook page likes (+273%) and Twitter hashtag use (+2,458%) but a negative trend (−92%) in the number of RFMC YouTube views. There was no statistically significant relationship between the number of views on the RFMC YouTube and RFMC associated SOME activity (*p* = 0.141). A significant relationship was shown between the number of Facebook page likes and the number of views on the RFMC YouTube (*p* = 0.037). Participants felt positively about the RFMC with recurring themes of; promotion, advocacy, public health, engagement, inspire, sharing, spreading information, feeling connected and general positive comments such as “enjoying tweets,” “great discussion.” Participants shared anecdotes, useful links, and book recommendations.

**Conclusion:** The RFMC has seen an increase in the amount of associated SOME activity despite having less viewers. This is most likely due to the few participants of the RFMC continuing the café discussions on SOME, particularly Twitter, and engaging outside of the RFMC. The RFMC has developed into a virtual community which is reducing occupational isolation for its participants.

## Introduction

Although approximately half of the global population lives in rural areas, less than a quarter of the total physicians' workforce work in rural areas (1). Demanding working conditions, substandard medical equipment and facilities, inadequate financial remuneration, inadequate opportunities for personal and professional growth, safety concerns, and lack of job opportunities for spouses and educational opportunities for children all contribute to the maldistribution of health professionals across the rural: urban divide (2). The provision of a stable and rewarding personal and professional environment has been cited as being key to a country's ability to recruit and retain health professionals in underserved areas (3). Social and professional isolation of rural healthcare professionals contributes to the perception that rural practice is difficult for professionals working in small rural communities, and to those students who aspire to a rural career (4).

Social media (SOME) provides healthcare professionals with tools to share information, to debate health care policy and practice issues, to promote health behaviors, to engage with the public, and to educate and interact with patients, caregivers, students, and colleagues (5). SOME refers to a variety of web-based applications which allow users to create and share content (6). This includes blogs/microblogs such as Twitter, internet forums such as Google groups, content communities such as YouTube, Flickr, and TikTok, and social networking sites such as Facebook or LinkedIn (7). The use of SOME is becoming increasingly prevalent in society particularly among individuals aged 45–54 (8).

A survey of more than 4,000 physicians conducted by the SoMesite QuantiaMD found that more than 90% of physicians use some form of SOME for personal activities, whereas only 65% use these sites for professional reasons (6, 9). SOME sites such as Facebook, Twitter, and YouTube are powerful symbols of a new generation of online tools and applications that foster user-generated content, social interaction, and real-time collaboration (10). There are manifold opportunities for professionals to use vast social networks to improve the wellbeing of patients and contribute to public health through the provision of high quality health information (10). The standards expected of doctors do not change because they are communicating through SOME rather than face-to-face or through other traditional media. However, using SOME creates new circumstances in which the established principles apply (11).

In response to a recognized need for increased communication between rural providers, the Rural Family Medicine Café (RFMC) was created. The RFMC is a SOME project created, originally on the Google Hangouts platform, in October 2015 to support and network doctors, students, and professors from around the world, interested in rural family medicine. The concept was first developed during the European Rural Conference in Croatia in 2015. Attending conferences gives rural professionals a sense of connection with likeminded colleagues however, this is in stark contrast to the realities of isolation they face when they return to rural communities or medical education which lacks a rural focus. The RFMC was developed to maintain this feeling of connectedness outside of the conference setting. With time, the concept spread to Facebook and Twitter using the hashtag “#ruralcafe” and the RFMC developed into an informal community for learning about rural medicine. It is a virtual place where people can meet together and share ideas, similar in setup to a coffee shop. The format is described in the Rural Cafe Manual; 15 steps to create a rural cafe, creating the opportunity for people to organize cafes locally (12). The RFMC was held monthly and usually included an international panel that discussed a topical issue in rural medicine. The café is then livestreamed to YouTube and others can join in the discussion by using #ruralcafe on Facebook and Twitter. Participants in the RFMC supported the creation of the World Rural Medicine Student Network, or Rural Seeds, which supports the engagements of healthcare students and communities with a rural background (13). In April 2016 “Rural Health Success Stories” was created as a result of networking between the founders of the RFMC and WONCA Rural South Asia [WoRSA (14)] (15). This led to the development of the hashtag “#ruralstories” with the aim to inspire, support, and reduce feelings of isolation among rural healthcare professionals. Similarly, the hashtag “#ruralwomenGP” was created to bring awareness of gender inequality in rural medicine.

This study aimed to investigate the impact of the RFMC on rural healthcare professionals and medical students interested in rural health. This study sought to quantify SOME activity associated with the RFMC and qualitatively analyse responses and discussions on the RFMC on Facebook, YouTube, and Twitter. It is hypothesized that the RFMC will be associated with qualitative evidence that it is seen as a good educational opportunity and a way to reduce feelings of professional and social isolation.

## Methods

Ethical approval for this study was granted by the Swansea University College of Human and Health Sciences and College of Medicine Research Ethics Committee reference #020117.

To assess the frequency of use of the hashtags associated with the RFMC, Twitter and Facebook searches were used to count the use of “#ruralcafe,” “#ruralwomenGP,” “#ruralGP,” “#ruralstories,” “#ruralmedicine” in each month from October 2015 when the RFMC started to October 2016. The topic of the RFMC in each month was also noted to investigate the correlation between the use of a particular hashtag and the topic. To establish a baseline measurement, the frequency of use in September 2015 of each hashtag was also measured. The number of followers on the RFMC Facebook page and the number of views of the RFMC YouTube videos in each month was also measured. With the data collected, a time series analysis was performed to compare the SOME activity in each month to the baseline measurement.

To qualitatively analyse the effect of the RFMC on feelings of isolation and lack of support, the content of tweets and Facebook posts using #ruralcafe, #ruralstories, and #ruralwomenGP were analyzed. Similarly, the comments on the RFMC YouTube videos were also analyzed. Comments were broadly classed as either positive or negative. Further data were collected by conducting a survey among RFMC participants. The survey was done using GoogleForms and the invitation was sent through the RFMC mailing list. Researchers did not know which participants responded to the survey thus the responses were randomized. The survey was opened from December 2016 to February 2017 and 54 responses were obtained. To account for participant bias, a question on whether the participant had viewed or participated in a RFMC was included in the survey but no participant answered this question. As a result, investigators do not know the level of experience each participant had with the RFMC. To supplement the information gathered from the survey, willing participants of the RFMC were invited for a semi-structured interview. Ten interviews were conducted with participants with the high engagement with the RFMC. The interview questions delved further into the participants' reasons and perceived benefits for participating, and issues that had been encountered. Questions used for the survey and semi-structured interview can be found in the Supplementary Material. Informed consent was obtained for participants who answered the survey and interview participants prior to collecting data.

### Inclusion Criteria

To be included in the study participants needed to have an interest in rural medicine, access to a computer and internet. Participants also needed to have an SOME account on FaceBook, Twitter, or Google+ and be able to communicate in English.

### Exclusion Criteria

Participants who were unable to communicate in English were not included in the study.

### Limitations

This study was limited by the necessity of internet access for the RFMC. The study involved the use of SOME platforms which may not be user friendly to all participants. The RFMC operates solely in English so participants who were unable to communicate in English could not participate. The hashtags #ruralmedicine, #ruralgp, #ruralwomenGP, and #ruralstories are general hashtags that are not specific to the RFMC. This may give a positive biased result which limits the reliability of study.

## Results

### Data Analysis

**Figure 1:**
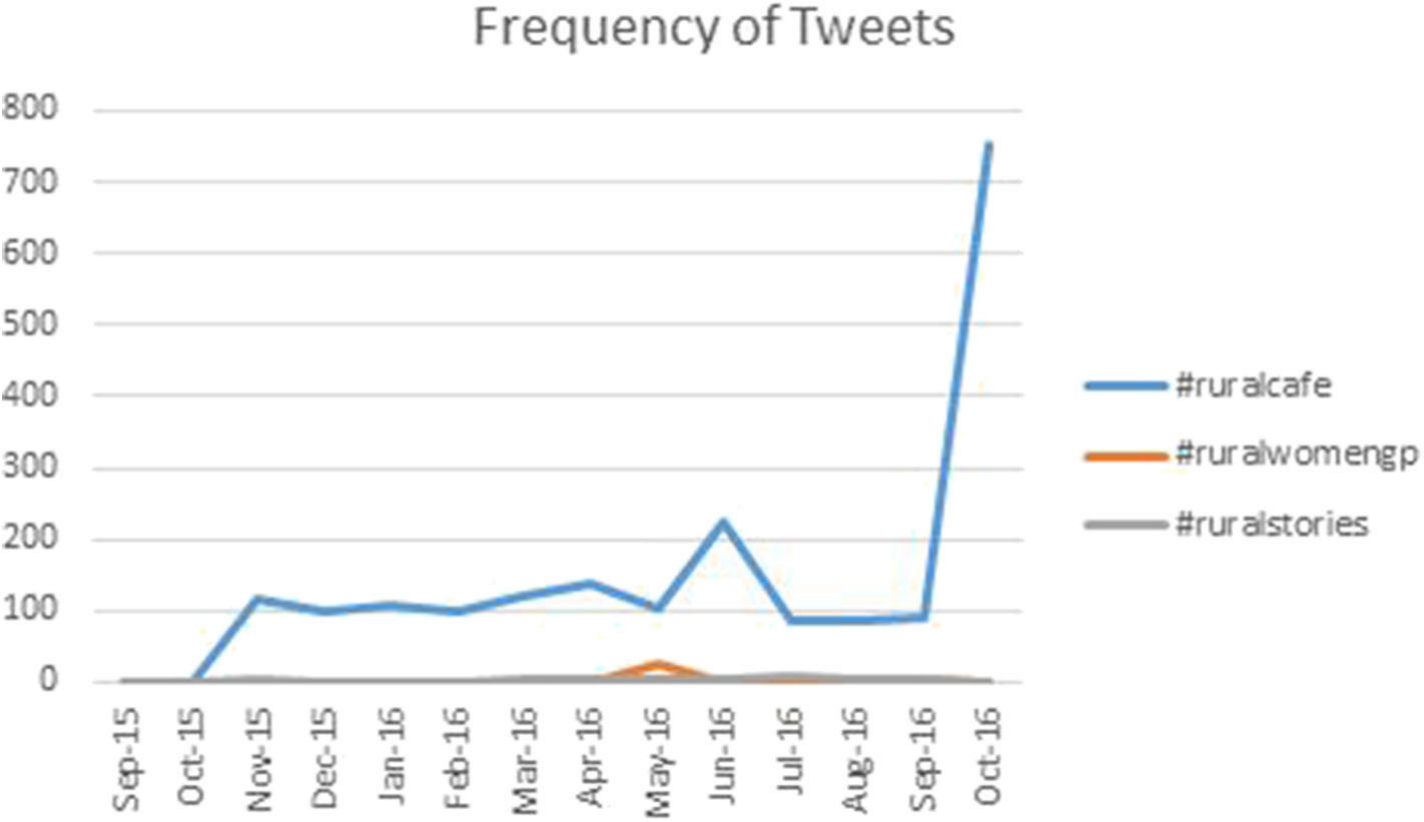
Line graph showing the frequency of tweets with hashtags #ruralcafe, #ruralwomengp, and #ruralstories.

Of the hashtags concerning the RFMC, #ruralcafe was used most frequently. Use of #ruralcafe took off in the month of October 2015 when the RFMC started, and remained relatively high with a spike in May 2016 and again October 2016 as shown in Figure 1. These spikes coincide with the Polaris conference and WONCA pre-conference respectively. A decline in the use of #ruralcafe was then seen in June 2016. The use of #ruralcafe then gradually increased with a peak in October 2016 as shown in Figure 1. #ruralwomengp had a peak in May 2016 when the project was first initiated but its use remained low thereafter. Rural Success Stories had the least frequent hashtag use.

**Figure 2:**
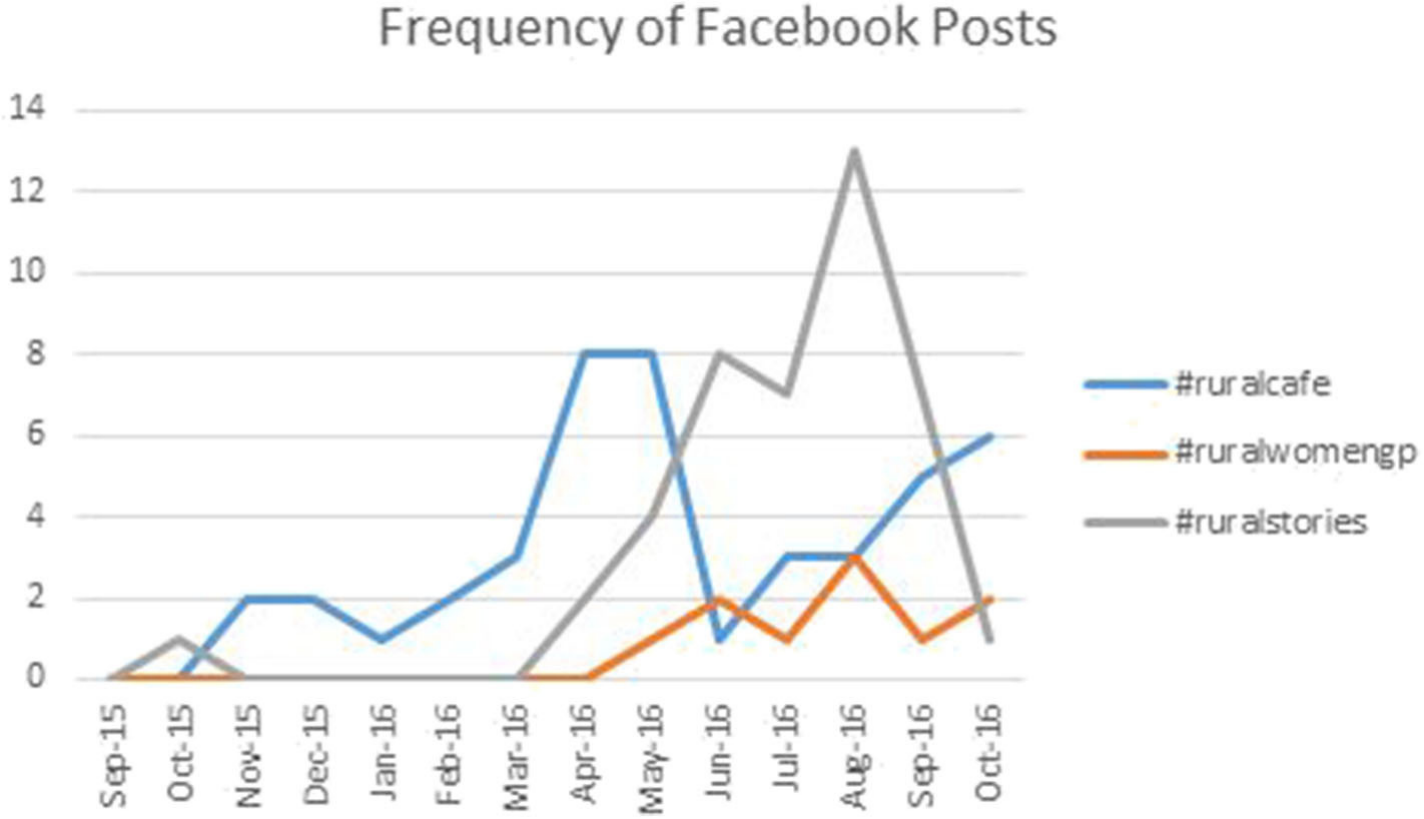
Line graph showing the frequency of Facebook posts with #ruralcafe, #ruralwomengp, and #ruralstories.

Facebook posts containing #ruralcafe were mainly announcements of past cafes and dates for upcoming cafes. Use of #ruralwomengp, as expected, was not seen until May 2016 when the project was initiated. Although used less frequently than #ruralcafe and #ruralstories, a gradual increase was seen. Between June 2016 and August 2016, #ruralstories was the most frequently used hashtag associated with the RFMC as shown in Figure 2. Facebook posts with #ruralstories were largely promotional with posts featuring quotes from stories that had been submitted. The engagement on Facebook did not follow the trends seen in use of the previously mentioned hashtags on Twitter.

**Figure 3:**
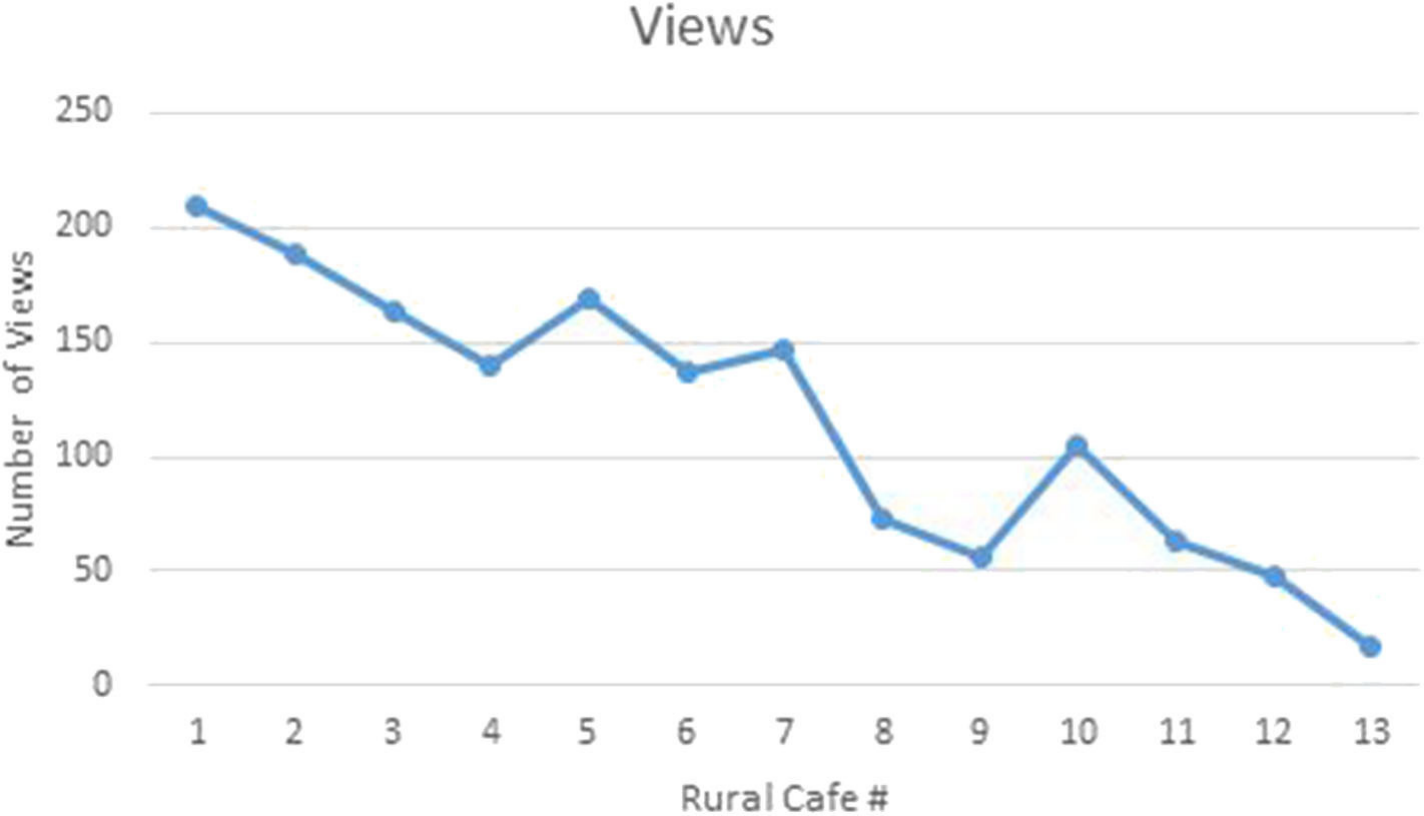
Line graph showing the number of YouTube views of each Rural Family Medicine Café.

**Figure 4:**
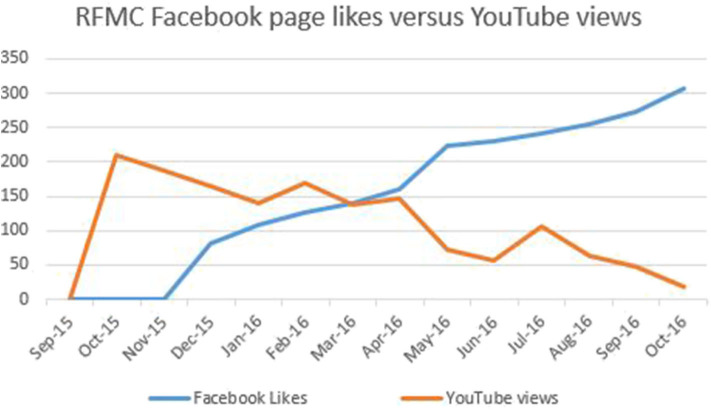
Line graph showing the number of RFMC Facebook page lives vs. the number views on the RFMC YouTube channel.

Each RFMC was livestreamed to YouTube which allowed persons to watch the video at their convenience. Since the first café, the number of views has gradually declined as shown in Figure 3. The highest number of views was achieved with the first café. Not included on the graph in Figure 3 are the views for the 14th RFMC held at the 2016 WONCA World Conference. The 14th RFMC was livestreamed *via* Facebook and had 407 views, the highest number of views for a RFMC. There was an inverse relationship between the number of Facebook page likes and the number of views on the RFMC YouTube channel with the number of views decreasing as the page likes increased. This is shown in Figure 4. There was a positive trend in the number of Facebook page likes (+273%) and Twitter hashtag use (+2,458%) but a negative trend (−92%) in the number of RFMC YouTube views. There was no statistically significant relationship between the number of views on the RFMC YouTube and RFMC associated SOME activity (*p* = 0.141). A significant relationship was shown between the number of Facebook page likes and the number of views on the RFMC YouTube (*p* = 0.037).

### Results of Participant Survey

53.7% of participants were female and 46.3% were male. The majority of participants, 61.1%, were between the ages of 26–39. The majority of participants were currently working in Family medicine either as qualified family doctors or trainees. 20.4% of participants were medical students. 51.9% of participants were currently practicing in the European continent. Thirteen percent of participants were practicing in Oceania, 13% in Asia, 11.1% in North America (the United States and Canada), and 9.3% in Africa.

The majority of participants used social networks occasionally for the purpose of learning with 27.8% using SOME frequently. When asked if they would use the RFMC for learning or professional development, 46.3% said they would probably use it, 22.2% said they would definitely use it, 16.7% would probably not use it, 11.1% were not sure, and 3.7% said they would definitely not use it for this purpose.

The majority of participants would recommend the RFMC to medical students interested in rural medicine. The majority of participants would also recommend the RFMC to their colleagues as a method for reducing professional isolation although this depended on how well the participant felt their colleague could navigate SOME. When asked about ease of access to the RFMC. 59.3% found it moderately difficult to access. 33.3% of participants found it easy to access the RFMC.

Looking at the demographic data of participants, those who identified as interns, residents, or junior doctors were more likely to use SOME and more likely to use hashtags associated with the RFMC. There was no correlation between age, gender, or continent of practice with SOME use. Of note in the survey data, none of the 54 participants volunteered data on what SOME platforms they were currently using.

Graphs illustrating the survey results can be found in the Supplementary Data.

### Qualitative Analysis

A thematic analysis using grounded theory was undertaken whereby authors analyzed separately the transcripts of interviews of a purposive sample of participants in the RFMC. The qualitative analysis was also used to assess participants' general response to the RFMC. Identified themes were discussed using a shared Google document group and themes refined. Interviewees were involved in triangulating the data through a process of consultation after the authors had completed their analysis and changes made to the conclusions after participant correction.

All interviewed participants in the RFMC had heard about the project either from a current participant or from hearing about it at conferences. Participants fit into two categories; regular attendees and *ad hoc* attendees. The main motivations for participating in the RFMC were; networking, international collaboration, sharing knowledge and experience, and participating in a forum with equal power of voice. Themes such as “support,” “education,” “morale,” “engagement,” “community,” and “network” were identified and grouped into five main themes which will be discussed in further detail later in this article. Benefits of participation noted by participants and elaborated by participant interviews included; reducing the feeling of isolation and improving social connections that could then be followed up on other platforms and/or face-to-face. Participants generally found the RFMC to be a source of stimulating learning through shared experience. For rural doctors in training, it was particularly beneficial to create links with more experienced doctors and found the experience empowering. Doctors in training found the informal cafe style facilitated networking and collaboration with more experienced doctors which would not necessarily occur as easily with face-to-face networking. The RFMC was noted to be of most benefit when it supplemented face-to-face connections. The RMFC also had a perspective of hope, which is key to move people and in this case especially rural health professionals (16).

## Discussion

The term #ruralcafe had the highest use in October 2016 due the 21st World WONCA conference. The pre-WONCA themed café was a platform to advertise the rural activities at WONCA and was also the 1 year birthday of the RFMC. At the WONCA conference, a live RFMC was held on November 5th, 2016. This achieved two things; (i) The RFMC was advertised to a larger audience and (ii) Participants were watching a discussion live and encouraged to tweet and post on Facebook. A number of participants expressed confusion on how to get involved in the RFMC so this second achievement may have removed a technological barrier to the café leading to increase SOME activity. Introducing the rural café at the 2016 World WONCA Conference in real time was also an easy way to introduce the use of SOME.

This suggests that the RFMC can be used in conjunction with promotion for conferences to improve sharing of information and networking. Additionally, the survey responses seem to suggest that an individual's previous experience with SOME was a better determinant for engagement that any other demographic. Qualitative analysis of interview responses collected identified 5 main themes; learning, impact on social and occupation isolation, recruitment and retention, rural advocacy, and networking and collaboration. Each of these themes will be discussed below.

### Learning in the RFMC

The RFMC is underpinned by an accepted theory of adult education called “social constructivism” whereby meaningful learning occurs in a social environment (17). In constructivism learning theory, knowledge is obtained by participants actively interpreting information from the experience of others and using that to build upon pre-existing knowledge. Constructivism relies on participants leading the learning process and having a previous knowledge base in order to integrate new experiences (18). The RFMC can be seen as a forum where educational opportunities are set within the workplace, this increased the validity and impact of participation particularly as it is led by the participant (18).

In social constructivism, knowledge is built within a social setting such as a class or in the case of the RFMC a virtual café. The dynamics of the group, such as the cultural background of each participant, thus contributes to the learning process (19). By having a variety of participants, less experienced participants learn through interacting with those who are more experienced while those with more experience learn from participants with a different background (20). One of the unique features of SOME is that you cannot only facilitate networking with like-minded peers but also with those from alternative backgrounds which creates diversity. The experience exchange between different ages and settings could bring a sense of empowerment and validation of rural health professionals. When a person could understand their reality, they can raise hypotheses about the challenge of that reality and look for a solution and explore what makes it possible to transform it (16).

The RFMC was reported to have improved collaborations among participants. It provided an opportunity to participate in scholarly activity, and teach and/or learn from international experiences. The extent to which these opportunities happen are dependent on how active a participant was with the RFMC however, there was potential to develop changes in attitude, knowledge and access to resources.

Key benefits of participating in RFMC highlighted by interviewees was that it was an opportunity to engage with others, share best practices and create learning opportunities in communities. As the knowledge shared in each cafe was based on lived experience, learning was “experientially driven” and this was felt to make learning relevant and valid as it was done within a real world context (21). The increased “reach” of a virtual cafe also increased the learning opportunities across international borders (22). The RFMC was seen as a useful tool for learning from others within the global context of medical practice. By participating in activities such as the RFMC, it was felt that one could increase knowledge of developments and changing trends thus alleviating the fear of rural healthcare professionals being “left behind.”

### RFMC Impact on Social and Occupation Isolation

Interviewed participants highlighted the geographical, professional, and technological isolation that rural healthcare professionals face. Due to these factors, rural healthcare professionals often work in an environment with minimal or no support leading to social and professional isolation. One unique aspect of social isolation highlighted by an interviewee is that isolation follows power differentials. There is a unique aspect to this among doctors due to their role as healers in the community. The interviewee referred to the “Aesculapian power.” Aesculapius was the Roman god of medicine and believed to hold the power of control over the quality of people's lives. In the doctor–patient relationship, Aesculapian power refers to the dynamic of power a doctor seems to have over their patient. The participant gave the example of the family physician in a rural community also holding other positions of leadership in sports or school councils. Effectively this maintains their “Aesculapian power” and can lead to social isolation when they are unable to step out of this role (23). The RFMC provided an environment where such hierarchies were not as socially relevant and so a participant could socialize outside of the Aesculapian power dynamic.

### Social Media and Rural Recruitment and Retention

SOME platforms can be used as a method of promoting careers in rural areas as well as promoting learning communities and events (9). Interviewed participants noted that the use of SOME platforms can help more experienced doctors engage with technological advances (24). This can in turn help in the recruitment of medical students/residents and facilitate rural exchanges. Anecdotally, there have been rural exchanges between participants of the RFMC as a result of networking on SOME. SOME can also help with rural recruitment and retention as it can lead to the development of social support mechanisms outside of the “Aesculapian power” dynamic previously mentioned (23). Students or young doctors are more likely to choose rural practice if they are aware of the opportunities of peer support (25).

SOME also has the positive impact of allowing personalities to be expressed which could aid in recruitment and the development of role models. It can also be used to increase the awareness of rural health professions. Many SOME platforms can be thought of as an open conversation that people can see without needing to actively participate in. Thus, SOME can act as a “shop window” for people interested in a career in rural health to see a variety of career possibilities.

### Social Media and Rural Advocacy

The theme of advocacy was common among interview responses and participants mentioned using SOME as a platform to advocate for the future of rural medicine among rural stakeholders, universities, and governments. The RFMC was recognized as a way of advocating for rural health issues, promoting rural practice, and demonstrating to potential rural health care professionals that peer groups could be formed even *in situ*ations of remote isolation. This is particularly useful for rural recruitment and retention as it can lead to the development of social support mechanisms (26). One interviewee reported that the RFMC could be used as a platform to advocate on behalf of rural communities as well as for the working conditions of rural healthcare professionals. Indeed the RFMC has been used to highlight issues that particularly affect rural communities including indigenous health and environmental health (27).

Interviewed participants were asked to describe what they felt the future of rural medicine would look like. Participants anticipated that government health policy makers would invest into “building” rural health in the long term. In the short term, more private initiatives would most likely be driving this development. The rural medicine of the future would have increased access to treatment and diagnostic tools through technology in practice. Thus allowing for increased research and integration of live broadcasts from conferences or use of other technological platforms at conferences. With this increased use of technology, more international collaboration is anticipated.

### Networking and Collaboration

The RFMC was used to encourage participants to attend conferences and meet face-to-face thus strengthening new interactions. The RFMC provided a “sense of community” and fostered connections that were followed up on other SOME platforms as well as face-to-face.

The RFMC was seen as a useful way of reducing both professional and social isolation. The RFMC was noted by one participant to provide an “alternative activity” in addition to providing peer support and meeting the needs of a “healer in a community” (23). The RFMC helped create links between more experienced doctors and less experienced doctors and students. This networking was reported to have relieved the anxiety of young doctors working in rural areas and also allowed for international collaboration in research.

An exemplar demonstration of this is the mentor–mentee program. This was a pilot project started by Rural Seeds which sought to pair newly qualified rural doctors with more experienced rural doctors. The pool of mentors and mentees was pulled from the Rural Seeds network which included participants of the RFMC. By having this connection, mentors, and mentees were able to build a relationship that led to international collaboration including presenting the experience of the mentor-mentee program at international conferences.

## Limitations

The RFMC process was hampered by the availability of good internet connections and issues around time zones. The use of SOME platforms highlighted the “digital divide” where there was an inequity of access to technology needed for large group discussion (24). Using a cafe style means learning is synchronous and this limits accessibility due to time zone differences. In addition to this, cafes are usually 2 h long which is a long time commitment. The RFMC was also unilingual and conducted in English. In the cafe style, it is important that education is developed by participants so that they see the value in the activity. Thus, the digital and lingual divide impacted participation particularly from low and middle income countries. As a result of this, rural organizations such as the Rural Doctors Association of Southern Africa (RUDASA), WONCA Rural Group South Asia (WoRSA WoRSA) Young Doctor's Movement Spice Route, and Rural Seeds Brazil (28) conducted local cafes or cafe style meetings that met the needs of their community of healthcare professionals in their native language.

One limitation which is unique to the SOME aspect of the RFMC is that there is potential to create an echo chamber effect (29). This can occur where the cafe participants are all people working in a similar field with a similar interest interacting with each other across multiple platforms. This can create a situation where certain beliefs are repeated, amplified, and upheld without criticism (29). With SOME this can also develop into a “celebrity culture” around more active participants whose word is given more value.

SOME is still an evolving process and requires active participation and, to some extent, risk taking to develop. As a result of this, communication *via* SOME can sometimes come across as superficial. It was acknowledged that SOME can augment other forms of learning but not replace them.

## Future Improvements

One of the challenges of the RFMC will be overcoming technical issues and the digital divide to improve and increase engagement and create a stable learning community that can continue to grow while keeping participants actively involved. Suggestion for improvement included curating content and creating thematic summaries with key learning points and links to resources that could be shared asynchronously. This indicated that improving ease of offline access to content from the cafe would be beneficial. A potential solution would be to develop the RFMC into a user-friendly, integrated platform which covers the specific needs of rural healthcare professionals. This would increase awareness of the RFMC, improve accessibility to content, and potentially introduce the use of SOME to more healthcare professionals and students.

The networking and collaboration opportunities created by the RFMC work well in conjunction with existing methods such as conferences, both local and international, and in formal educational settings. While locally organized cafes do have their benefit, the greatest networking capability is through introducing the concept of the rural cafe to large groups. In conjunction with live cafes, the RFMC platform could be used as part of medical education which would improve engagement with the virtual community.

## Conclusion

Social networking promotes communication as professionals, in our case healthcare professionals, with relatively common backgrounds and interests are able to interact. In other words, health care providers can communicate with other health professionals, patients, etc. Also specific social networking websites allow for the users to communicate in groups, and so the communication with different stakeholders is more practical.

The RFMC was a project which aimed to improve engagement of healthcare professionals, medical students, and rural community members *via* SOME. The RFMC has seen an increase in SOME activity particularly when used in conjunction with face-to-face meetings such as conferences. Analysis of the data gathered in this study suggests that this may be due to a handful of RFMC participants continuing discussions from the cafe on SOME, particularly Twitter, as well as in face-to-face discussions. Results from the survey suggest that the best indicator for a participant's likelihood to use SOME and RFMC related hashtags was their prior experience with SOME. By continuing RFMC discussions both on- and off-line, RFMC participants can engage with those who have previously not engaged with SOME thus building the virtual RFMC community. SOME and other new technologies have become more popular as they are a more cost-effective method of communication and collaboration. This has been demonstrated by the increased use of telecommunication and tele-health modalities due to the COVID-19 pandemic and restrictions on face-to-face meetings.

RFMC participants generally felt positively about the RFMC and benefits of participation identified were; the RFMC as an informal learning environment, reduction in social and occupational isolation, potential to improve recruitment and retention of rural healthcare professionals, potential to advocate for rural healthcare professionals and rural communities, and a method of networking and collaboration that reduces geographical and financial limitations. Limitations identified were that the RFMC is monolingual which created a language barrier and that participation relied on access to a device and stable internet connection. There were also difficulties around organizing a meeting with participants across several time zones. As a result of these limitations, several organizations held local RFMCs.

Potential areas of improvement identified were; curating content and creating thematic summaries from each cafe to facilitate offline access to content from the cafe. A potential solution would be to develop the RFMC into a user-friendly, integrated platform which covers the specific needs of rural healthcare professionals. This could facilitate the RFMC being used in conjunction with face-to-face settings for the purpose of medical education which would improve engagement with the virtual community.

The RFMC has grown into a virtual community and informal learning environment. This has helped to reduce feelings of social and occupational isolation among participants in addition to providing opportunities for networking and collaboration. As has become evident with the COVID19 pandemic, the training and the ongoing support for new technologies should be a high priority especially in order to provide qualified services in rural and difficult to access areas.

## Data Availability Statement

All datasets generated for this study are included in the article/Supplementary Material.

## Ethics Statement

Ethical approval for this study was granted by the Swansea University College of Human and Health Sciences and College of Medicine Research Ethics Committee reference #020117.

## Author Contributions

The RFMC project was conceptualized and started by MF. The abstract of this paper was written by AW. The introduction and background of the project was done by MF, MB, and MK. Data collection including formulation, distribution, and analysis of the survey used in this research was done by AW, BS, and MF. There was a significant contribution to this section by JS-J. The conclusion was written by MK and AW. Overall editing and coordination of writers was done by AW. All authors were involved in qualitative analysis of interview responses and identifying recurring themes.

## Conflict of Interest

The authors declare that the research was conducted in the absence of any commercial or financial relationships that could be construed as a potential conflict of interest.
