# Supplementary material for: The Rural Family Medicine Café Project: A Social Media Strategy to Reduce Occupational Isolation and Improve Support for Rural Healthcare Professionals
Source: Front Public Health. 2020 Nov 19;8:595255. doi: 10.3389/fpubh.2020.595255 (PMC7710660; doi:10.3389/fpubh.2020.595255)
Supplement: Supplementary file 1 [file Data_Sheet_1.DOCX]

Rural Family Medicine Café Project Supplementary Material

# Supplementary Figures

**
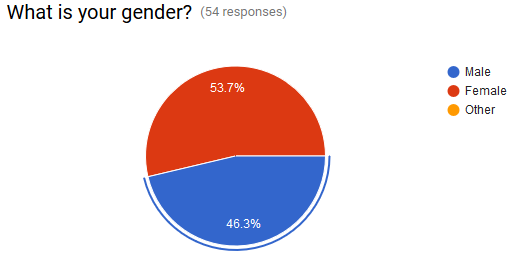
**

**Supplementary Figure 1:** pie chart showing the gender identity of RFMC participants

**
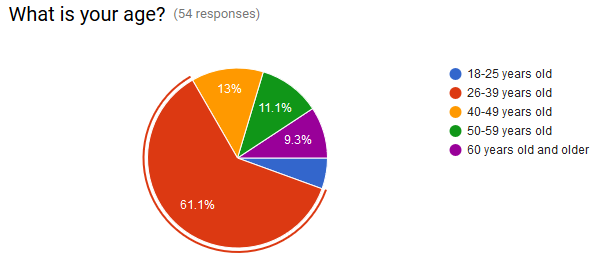
**

**Supplementary Figure 2:** pie chart showing the age category of RFMC participants

**
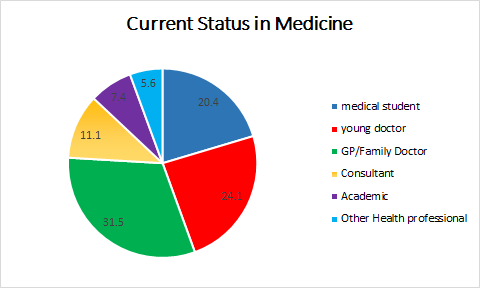
**

**Supplementary Figure 3:** pie chart showing the stage of training of RFMC participants

**
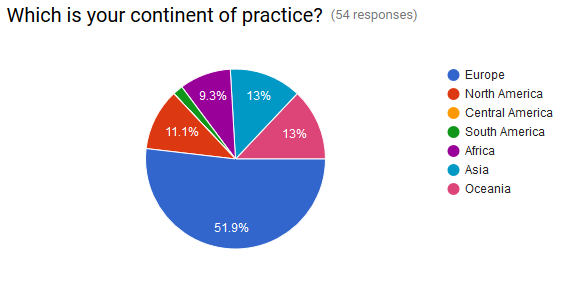
**

**Supplementary Figure 4:** pie chart showing the continent of practice of each participant

**
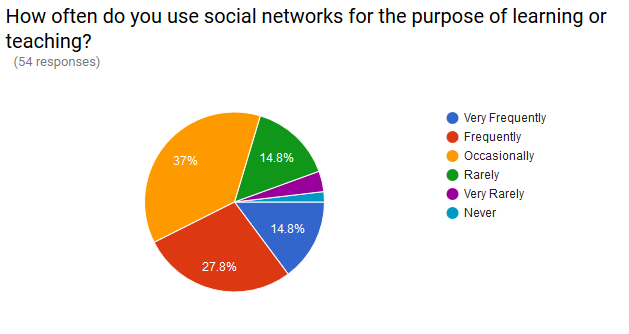
**

**Supplementary Figure 5:** pie chart showing how frequently participants use social media for the purpose of learning or teaching

**
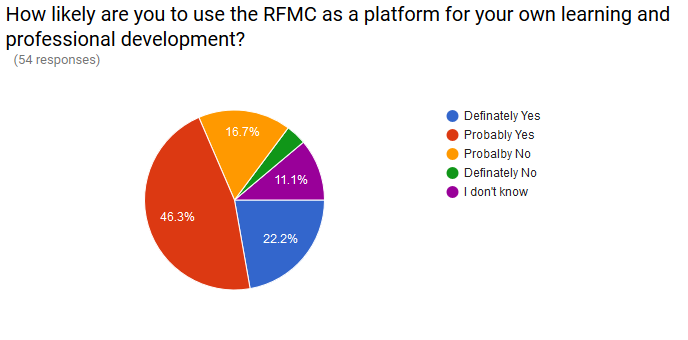
**

**Supplementary Figure 6:** pie chart showing how likely each participant was to us the RFMC for their own learning and professional development

**
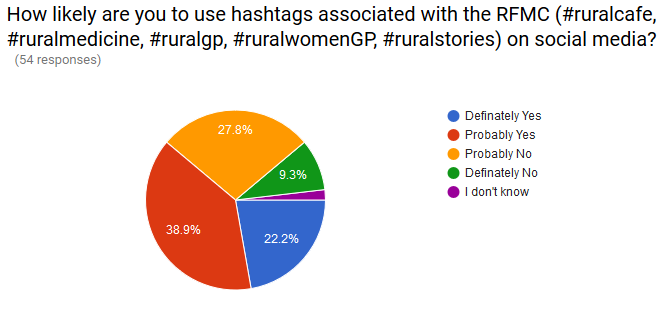
**

**Supplementary Figure 7:** pie chart showing how likely participants were to use hashtags associated with the RFMC on social media

**
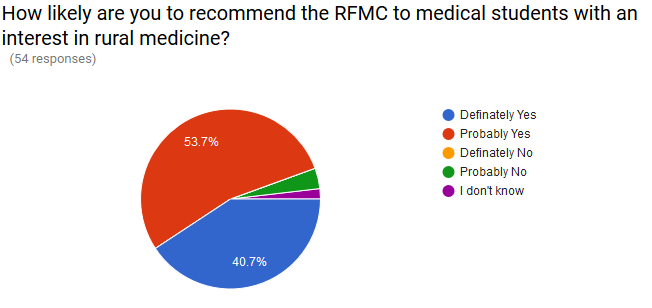
**

**Supplementary Figure 8:** pie chart showing proportion of RFMC participants who would recommend the RFMC to medical students interested in rural medicine

**
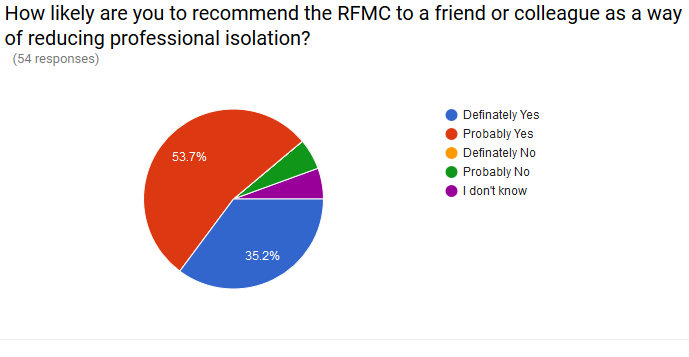
**

**Supplementary Figure 9:** pie chart showing the proportion of RFMC participants that would recommend the RFMC to colleague to reduce professional isolation

**
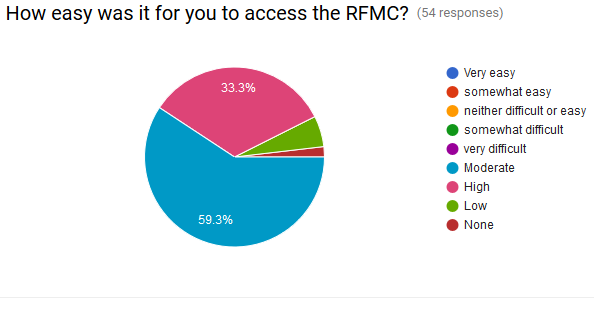
**

**Supplementary Figure 10:** pie chart showing how participants found access to the RFMC

# Supplementary Tables

Supplementary Table 1: Data on Frequency of Hashtag use on Twitter

| **Month** | **Hashtag** | | | |  |
| --- | --- | --- | --- | --- | --- |
|  | **#ruralcafe** | **#ruralwomengp** | **#ruralstories** | **#ruralmed** | **total** |
| Sep-15 | 0 | 0 | 1 | 30 | 31 |
| Oct-15 | 0 | 0 | 1 | 13 | 14 |
| Nov-15 | 116 | 0 | 3 | 41 | 160 |
| Dec-15 | 99 | 0 | 0 | 21 | 120 |
| Jan-16 | 106 | 0 | 0 | 34 | 140 |
| Feb-16 | 98 | 0 | 0 | 24 | 122 |
| Mar-16 | 121 | 0 | 2 | 17 | 140 |
| Apr-16 | 139 | 0 | 3 | 33 | 175 |
| May-16 | 103 | 26 | 3 | 46 | 178 |
| Jun-16 | 224 | 1 | 2 | 37 | 264 |
| Jul-16 | 85 | 4 | 7 | 14 | 110 |
| Aug-16 | 87 | 4 | 3 | 17 | 111 |
| Sep-16 | 89 | 4 | 2 | 47 | 142 |
| Oct-16 | 751 | 1 | 0 | 41 | 793 |

Supplementary Table 2: Data on Frequency of Hashtag use on Facebook

| **Month** | **Hashtag** | | | |
| --- | --- | --- | --- | --- |
|  | #ruralcafe | #ruralwomengp | #ruralstories | #ruralmed |
| Sep-15 | 0 | 0 | 0 | 2 |
| Oct-15 | 0 | 0 | 1 | 1 |
| Nov-15 | 2 | 0 | 0 | 3 |
| Dec-15 | 2 | 0 | 0 | 1 |
| Jan-16 | 1 | 0 | 0 | 1 |
| Feb-16 | 2 | 0 | 0 | 0 |
| Mar-16 | 3 | 0 | 0 | 0 |
| Apr-16 | 8 | 0 | 2 | 0 |
| May-16 | 8 | 1 | 4 | 1 |
| Jun-16 | 1 | 2 | 8 | 1 |
| Jul-16 | 3 | 1 | 7 | 6 |
| Aug-16 | 3 | 3 | 13 | 5 |
| Sep-16 | 5 | 1 | 7 | 3 |
| Oct-16 | 6 | 2 | 1 | 3 |
| Nov-16 | 13 | 3 | 1 | 0 |

Supplementary Table 3: Data on Number of RFMC Facebook Page Likes

| **Date** | **Likes** |
| --- | --- |
| Sep-15 | 0 |
| Oct-15 | 0 |
| Nov-15 | 0 |
| Dec-15 | 82 |
| Jan-16 | 108 |
| Feb-16 | 127 |
| Mar-16 | 141 |
| Apr-16 | 160 |
| May-16 | 224 |
| Jun-16 | 230 |
| Jul-16 | 241 |
| Aug-16 | 256 |
| Sep-16 | 273 |
| Oct-16 | 306 |

Supplementary Table 4: The number of RFMC Facebook page likes compared to RFMC YouTube channel views

| Month | Facebook likes | YouTube views |
| --- | --- | --- |
| Sep-15 | 0 | 0 |
| Oct-15 | 0 | 209 |
| Nov-15 | 0 | 188 |
| Dec-15 | 82 | 164 |
| Jan-16 | 108 | 140 |
| Feb-16 | 127 | 169 |
| Mar-16 | 141 | 137 |
| Apr-16 | 160 | 147 |
| May-16 | 224 | 73 |
| Jun-16 | 230 | 56 |
| Jul-16 | 241 | 105 |
| Aug-16 | 256 | 63 |
| Sep-16 | 273 | 48 |
| Oct-16 | 306 | 17 |

Supplementary Table 5: Data on the number of views, likes, shares, and comments on each RFMC

| Café | Views | Likes | Shares | Comments |
| --- | --- | --- | --- | --- |
| 1 | 209 | 6 | 6 | 0 |
| 2 | 188 | 4 | 2 | 3 |
| 3 | 164 | 3 | 4 | 3 |
| 4 | 140 | 2 | 1 | 1 |
| 5 | 169 | 4 | 5 | 0 |
| 6 | 137 | 4 | 0 | 0 |
| 7 | 147 | 3 | 1 | 0 |
| 8 | 73 | 0 | 0 | 3 |
| 9 | 56 | 3 | 0 | 0 |
| 10 | 105 | 3 | 1 | 1 |
| 11 | 63 | 0 | 1 | 0 |
| 12 | 48 | 1 | 1 | 0 |
| 13 | 17 | 0 | 0 | 0 |

# Rural Family Medicine Café Participant Survey

INTRODUCTION

Young doctors and medical students commonly practice in rural areas where medical decisions become monopolized by a single person’s actions. Isolation and little access to expert knowledge or the opinions of those in secondary or tertiary care are everyday problems faced by rural healthcare professionals. Social Media (SoMe) may offer solutions to these problem due to the widespread utilization of social media and its power to reach a wide audience.

The Rural Family Medicine Café (RFMC) is a SoMe project created to support and network doctors, medical residents and students, professors and people interested in rural medicine from all over the world. The RFMC is held monthly with an international panel that discusses a topical issue in rural medicine and it is a place where people gather like in a Café, drink their coffee discussing about various topics concerning medicine, share ideas and create awareness. Discussions are done without distinction between academic background (professors, students, young doctors and experienced doctors) and every participant has the same power of voice.

INFORMED CONSENT

I was invited to participate in the study "RFMC Research Project: Can the Rural Family Medicine Cafe Reduce Occupational Isolation and Improve Support for Rural Healthcare Professionals?" written by Amber Wheatley (BSc Swansea University Medical School, UK), Mayara Floss (Federal University of Rio Grande, Brazil), Jo Scott-Jones (MD, Auckland University Hon Senior Lecturer), Maria Bakola (MD, Family Medicine Physician/ General Practitioner, Derviziana Primary Care Health Center and General Hospital of Ioannina " G.Hatzikosta" , Ioannina, Greece) Bianca Silveira (Escola de Saude Publica, Florianopolis)and Maria Kampouraki ( MD, Primary Care Practice of Pompia, Crete).

In the survey, I will answer a questionnaire in electronic format, with open and closed questions relating to my demographic information and my academic background. My answers will help researchers to investigate the impact of the RFMC in generating interest and activity in rural medicine among medical students and young doctors by using social media.

I am aware that I can refuse to participate or withdraw at any time of the questionnaire and the research project without the need to explain and therefore will not suffer any kind of damage or injury in the present or in the future. I can also choose not answer any question.

This research does not pose any direct risks to my health, welfare or dignity

The benefits of this research will be the expansion of knowledge about quantifying social media activity associated with the RFMC and qualitatively analysing responses to the RFMC discussions on Facebook, YouTube and Twitter.

I was informed that confidentiality and anonymity are secured and guaranteed, that the data will be recorded and used only for scientific purposes, that it is the researcher's responsibility to guard them, that access to data will only be made by researchers and that the dissemination of research may occur in the form of research reports, articles in scientific journals, scientific or professional events, among others. My email address will be stored only to track survey completion. The data will be reported only in the aggregate and no individual will be identified.

I will have no cost to participate in this study. I agree to voluntarily participate in this study and declare that all my questions were answered. While agreeing to participate, I am not giving up my right to withdraw from the study in future

If I want to ask any other question or request any clarification I can contact the researchers.

* Required

1. Do you agree with the above terms? * Mark only one oval.

Yes

No - Skip to question 14

Participant Information

This information is collected for the purpose of data analysis and will be kept anonymised

2. What is your gender? * Mark only one oval.

Male

Female

Other:

3. What is your age? * Mark only one oval.

18-25 years old

26-39 years old

40-49 years old

50-59 years old

60 years old and older

4. Which is your current status in medicine? * Mark only one oval.

Medical student

Young doctor (5 years or less since qualifying)

GP/Family Doctor

Consultant

Academic

Other Health Professional

5. Which is your continent of practice? * Mark only one oval.

Europe

North America

Central America

South America

Africa

Asia

Oceani

6. How often do you use social networks for the purpose of learning or teaching? * Mark only one oval.

Very Frequently

Frequently

Occasionally

Rarely

Very Rarely

Never

7. Do you use any other online platforms for networking, teaching or learning? If so what platforms do you use?

Questionnaire

8. Have you ever attended or watched a Rural Cafe? Mark only one oval.

Yes, attended

Yes, watched

No

9. How likely are you to use the RFMC as a platform for your own learning and professional development? * Mark only one oval.

Definately Yes

Probably Yes

Probalby No

Definately No

I don't know

10. How likely are you to use hashtags associated with the RFMC (#ruralcafe, #ruralmedicine, #ruralgp, #ruralwomenGP, #ruralstories) on social media? * Mark only one oval.

Definately Yes

Probably Yes

Probably No

Definately No

I don't know

11. How likely are you to recommend the RFMC to medical students with an interest in rural medicine? * Mark only one oval.

Definately Yes

Probably Yes

Definately No

Probably No

I don't know

12. How likely are you to recommend the RFMC to a friend or colleague as a way of reducing professional isolation? Mark only one oval.

Definately Yes

Probably Yes

Definately No

Probably No

I don't know

13. How easy was it for you to access the RFMC? * Mark only one oval.

Very easy

somewhat easy

neither difficult or easy

somewhat difficult

very difficult

Questionnaire feedback

The data collected from this research will be used to assess the use of the RFMC in preventing occupational isolation and improving support of rural healthcare professionals. The data collected will be anonymised and kept secured. Data will only be used for scientific purposes and may be used to write research reports, articles in scientific journals or professional events. Your email address will be stored by the principal investigator for the purpose of tracking survey completion. The data will be reported only in the aggregate and no individual will be identified.

You are free to withdraw from this study at any point without consequence. If you want to ask any other question or request any clarification I can contact the researchers.

The Rural Family Medicine Cafe team would like to sincerely thank you for your participation in our research project. If you wish to withdraw from the study or have any questions, you can contact Amber Wheatley at [vashwheatley@hotmail.com](mailto:vashwheatley@hotmail.com), Bianca Silveira at [biancansilveira@gmail.com](mailto:biancansilveira@gmail.com) or Mayara Floss at floaramoss@hotmail.com

14. Please, if you want, add comments and suggestions to improve this questionnaire. *

# Semi-structured Interview Questions

RFMC as a platform for learning

1)    How did you get involved in the RFMC?

2)    What was your reason for joining the RFMC?  3)    How long would you consider yourself “active” in the RFMC?

4)    What do you think you have gained from the RFMC?

RFMC and social media

1)    What do you think about the use of social media among healthcare professionals?

2)    What do you think the benefits of using social media are in rural healthcare? E.g. hashtags, tweets, YouTube or Facebook.

3)    What problems do you think can be experienced using social media?

RFMC for medical education

1)    What platforms do you normally use for your medical education?

2)    How do you think medical education, particularly rural medical education, is changing with social media becoming more prevalent?

3)    How do you think social media can be used to encourage those interested or active in rural health?

4)    What are your thoughts on the RFMC being used as a learning platform?

RFMC as a way of reducing professional isolation

1)    What do you think about professional isolation in rural health? What does that term mean to you?

2)    What do you think healthcare professionals can do to overcome occupational isolation in rural medicine?

3)    What are your thoughts on the impact, if any, of the RFMC on rural healthcare professionals?

Feasibility of RFMC Participation?

1)    What challenges, if any, have you experienced as a participant in the RFMC.

2)    What do you think the future of rural medicine will look like?

3)    What role do you think the RFMC will play in the future of rural medicine?

# 4)    The RFMC aims to target rural healthcare professionals, what are your thoughts on this aim?
